# EANM/EARL harmonization strategies in PET quantification: from daily practice to multicentre oncological studies

**DOI:** 10.1007/s00259-017-3740-2

**Published:** 2017-06-16

**Authors:** Nicolas Aide, Charline Lasnon, Patrick Veit-Haibach, Terez Sera, Bernhard Sattler, Ronald Boellaard

**Affiliations:** 1 Nuclear Medicine Department, University Hospital, Caen, France; 2 Inserm U1086 ANTICIPE, Caen University, Caen, France; 3 Nuclear Medicine Department, François Baclesse Cancer Centre, Caen, France; 4 Department of Nuclear Medicine and Department of Diagnostic and Interventional Radiology, University Hospital Zurich, Zurich, Switzerland; 5 Joint Department Medical Imaging, University Health Network, University of Toronto, Toronto, Canada; 6 Nuclear Medicine Department, University of Szeged, Szeged, Hungary; 7 Department of Nuclear Medicine, University Hospital of Leipzig, 04103 Leipzig, Germany; 8 Department of Nuclear Medicine and Molecular Imaging, University of Groningen, University Medical Center Groningen, Groningen, The Netherlands; 9 Department of Radiology and Nuclear Medicine, VU University Medical Center, Amsterdam, The Netherlands

**Keywords:** PET/CT, SUV, MATV, EARL accreditation, Harmonization, EORTC, PERCIST, Deauville score

## Abstract

Quantitative positron emission tomography/computed tomography (PET/CT) can be used as diagnostic or prognostic tools (i.e. single measurement) or for therapy monitoring (i.e. longitudinal studies) in multicentre studies. Use of quantitative parameters, such as standardized uptake values (SUVs), metabolic active tumor volumes (MATVs) or total lesion glycolysis (TLG), in a multicenter setting requires that these parameters be comparable among patients and sites, regardless of the PET/CT system used. This review describes the motivations and the methodologies for quantitative PET/CT performance harmonization with emphasis on the EANM Research Ltd. (EARL) Fluorodeoxyglucose (FDG) PET/CT accreditation program, one of the international harmonization programs aiming at using FDG PET as a quantitative imaging biomarker. In addition, future accreditation initiatives will be discussed. The validation of the EARL accreditation program to harmonize SUVs and MATVs is described in a wide range of tumor types, with focus on therapy assessment using either the European Organization for Research and Treatment of Cancer (EORTC) criteria or PET Evaluation Response Criteria in Solid Tumors (PERCIST), as well as liver-based scales such as the Deauville score. Finally, also presented in this paper are the results from a survey across 51 EARL-accredited centers reporting how the program was implemented and its impact on daily routine and in clinical trials, harmonization of new metrics such as MATV and heterogeneity features.

## Background: The need to harmonize procedures

### Metrics frequently used in PET/CT quantification

Quantification of whole body oncology FDG PET/CT studies is mainly performed using standardized uptake values (SUVs). SUVs are computed with the following equation:

$$ SUV=\frac{\mathrm{Activity}\ \mathrm{in}\ \mathrm{tumour}\ \left( Bq/ cc\right)}{\mathrm{Injected}\ \mathrm{activity}\ (Bq)}\times \mathrm{weig} ht\ (g) $$

The activity in the tumor can be derived by using, for example, the maximum uptake in the tumor, providing SUV_max_, or by using the average over a region of interest, SUV_mean_. If the region of interest is given by a 1 mL sphere positioned to yield the highest value in the tumor, SUV is referred to as SUV_peak_. The injected activity represents the net administered FDG activity, corrected for decay and residual activities in the administration system or syringe. Patient weight is still most commonly used as the normalization factor in the equation. However, given that hardly any FDG is taken up by fat and that antineoplastic treatments can affect the patient’s weight, the lean body mass (LBM) has been recommended instead of weight. LBM is usually based on weight and height measurements, though it has been shown that it could be extracted from the low-dose CT component of the PET/CT acquisition [1–3]. Further details on LBM evaluation can be found in the last section of this review, together with other suggested improvements in SUV calculations.

**Fig. 1:**
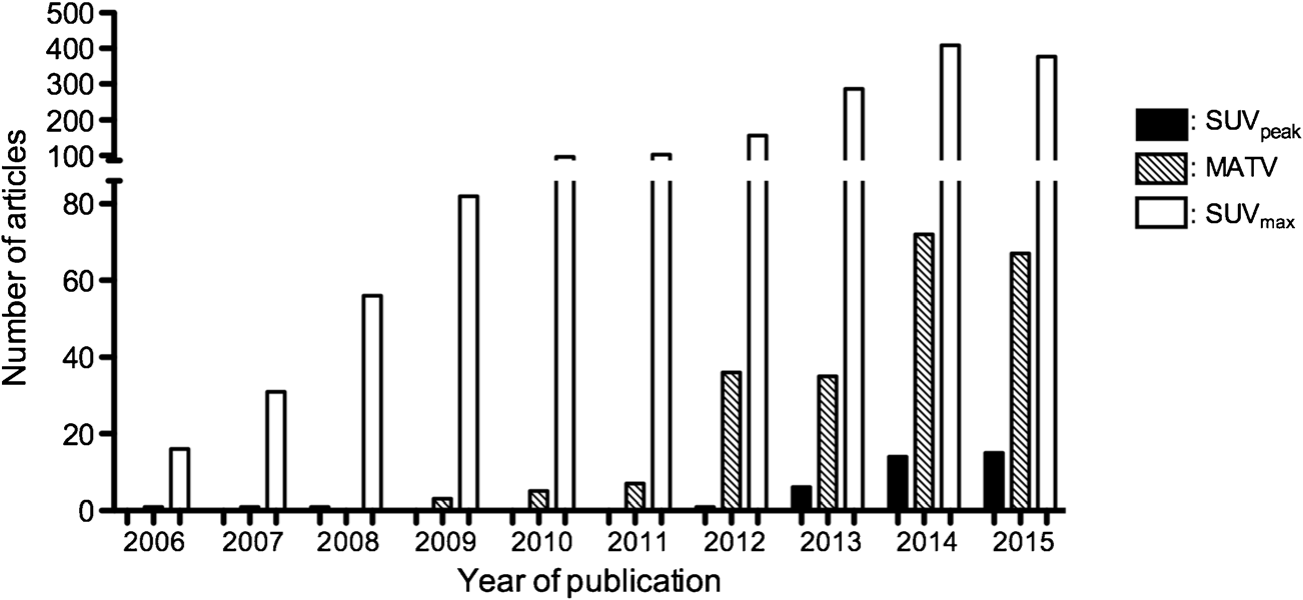
Number of articles reporting the use of MATV, SUV_max_ and SUV_peak_ as a function of year of publication. Articles were identified by Medline search with the following keywords: (MTV OR MATV AND PET), (SUV_max_ AND PET) or (SUV_peak_ AND PET). Only human studies were included

Recently there is increasing interest in deriving the metabolic active tumor volumes (MATVs) and total lesion glycolysis (TLG) metrics. MATV can be obtained by delineating the tumor using, for example, a 41% of SUV_max_ isocontour threshold as per EANM guidelines [4, 5], or by advanced algorithms including information on gradients or the background surrounding the tumor. The frequency of MATV usage, irrespective of the methodology used for tumor contouring, is shown in Fig. 1.

MATV has gained a lot of interest as a pre-treatment prognostic tool in various cancer types, but can be hampered by the same errors as for SUVs, with variability in tumor delineation methodology being one of the major sources of variability. Delineation of MATVs is also useful for radiotherapy planning in various cancers including non-small cell lung cancer (NSCLC) [6]. The impact of PET imaging parameters on automatic tumor delineation for radiotherapy planning has been well documented [7–9], prompting the need for an improved and standardized delineation methodology. Also, though recent studies in non-Hodgkin lymphoma (NHL) have shown high MATV to be predictive for overall survival [10], widely disparate cut-off values were found, fuelling the ongoing reflexions on the need to standardize the quality of PET images and the delineation methodology.

Fig. 1 shows the frequency of use of the different SUV metrics and MATV as of December 2016.

SUV and MATV can be used as biomarkers for diagnostic or prognostic purposes, but their main use is therapy monitoring of antineoplastic treatments. The use of these metrics to evaluate response to a given treatment is based on the fact that the observed changes in tumor uptake are greater than that due to inherent statistical fluctuations. In that setting, recent test-retest studies have shown repeatability of SUV measurements better than those published in former generation PET systems, including standalone PET. A specific issue is the variability in SUV calculated by different software packages, as was pointed out by, among others, Pierce et al. [11].

### Issues related to quantification in PET/MR

In the last five years, cross-modality hybrid PET imaging combined with MRI has started to enter the clinical arena. Both sequential [12] and integrated systems [13, 14] are available using different PET signal detection technologies. MRI offers superior soft tissue contrast depiction over CT, where more dense structures like bone are resolved best. For the quantitative validity of the PET measurements – i.e. the correct determination of the aforementioned quantitative parameters – it is essential that the concentration of activity in respective lesions, volumes and sub-volumes (Bq/cm^3^) be determined as accurately as possible. Therefore, the attenuation and scatter of the 511 keV photons, until they reach the detector system, need to be involved in the reconstruction of the emission data set. Attenuation of photons is mainly determined by the electron density of the material they travel trough and interact with. With CT this electron density can be directly obtained by using the CT transmission volume data set after a (bi-linear) calibration of the linear attenuation coefficients. In the case of PET/MR, the attenuation correction (AC) is derived from a dedicated MR-AC protocol. In most cases the obtained MR image is first segmented into two or three tissue classes. The segmented tissue classes are assigned a constant linear attenuation coefficient and the so-constituted segmented μ-map is used for attenuation correction of the emission data. Despite extensive research in this field, these algorithms suffer from being insufficient to detect bone and air. Moreover, often the lungs are assumed to be uniform and not all air pockets (nasal cavities) are properly segmented. In their recent implementations most of the vendors use ultrashort- and zero echo time MR sequences to detect bone (in certain body areas, e.g. the head) and, thus, improve the performance of the tissue class segmentation [15, 16]. These methods are combined with methods of μ-map generation from MR data that use structural (i.e. T1- or T2 weighed) MR data sets in combination with CT-atlas based information of a particular part of the body to generate a more realistic map of linear attenuation coefficients, including bone [17–21]. In recent research settings, neuronal network approaches are employed to train algorithms using real CT data to learn, generating continued valued maps of LACs on the basis of structural MR data sets. Using these methods and depending on the body compartment, the accuracy of the PET measurement in hybrid PET/MRI settings now reaches the order of accuracy of that in PET/CT settings. Yet, in particular cases (pediatric, metal implants, ports, etc.) inaccurate attenuation maps may still occur. All the hardware in the path of the gamma rays needs to be taken into account, as it also attenuates the PET signal. The (flexible or rigid) MR signal receiver coils and the patient table are either implemented by CT-measured maps of LACs or designed in a way that the attenuation of the PET signal by this material is negligible. Most of the harmonization procedures of quantitative PET, as known from PET/CT, are based on the measurement of known phantom structures filled with watery solutions of radioactivity containing different fillable sub-volumes and, thereby, representing known activity concentrations in volumes of different sizes in an either cold or hot background. Firstly, being constructed mainly of plastic, the structure of those phantoms cannot be detected sufficiently by MRI. Secondly, large volumes of water in the MRI field of view cause major distortions of the MR signal. This topic has been addressed by searching for alternative liquids to fill the phantom [22]. Current approaches to use activity fillable phantoms in hybrid PET/MRI, however, employs the implementation of CT-generated μ-maps of the particular phantom to account for the attenuation of the PET signal. Thus, inter-system quantitative comparisons give just the comparability of the quantitative performance of the PET detector systems. If the clinical settings for attenuation correction – i.e. the MR-based μ-map – is used for attenuation correction of phantom measurements, considerable deviations of accuracy of the PET measurement are found [23, 24].

The latest generation of hybrid PET/MRI systems is capable of Time Of Flight (TOF) PET signal detection [14]. This information can be used for simultaneous reconstruction of activity and attenuation [25, 26], which might enable further improvement in the quantitative accuracy of PET/MR studies and/or the mitigation of MR-AC related PET image artifacts.

There are several clinical implications arising from the differences in PET-quantification between PET/CT and PET/MR. Generally it is known that there is an underestimation based on the above described Dixon-based attenuation method. This underestimation is especially evident close to bone [27]. Diagnostically the problem here is that detection of lesions in or close to a bony structure can be impaired. This naturally leads to possible underestimation of the disease extent, especially in oncological diseases with preference to bone metastases (e.g. breast cancer, prostate cancer ,etc.) and thus inadequate therapy decisions.

Moreover, comparability between follow-up studies in PET/MR can be difficult, not only on the same system but also when considering different PET/MR systems [23]. After therapy, glucose-utilization of tumorous lesions usually decreases, thereby indicating therapy response, even in cases where the lesion’s size does not fulfill the criteria of partial response. However, in cases of incorrect underestimation of a lesion’s FDG-uptake, lesions might appear as no longer having elevated uptake, whereas they in fact are still FDG-avid. Here again, consecutive therapy misclassification cannot be excluded in such cases.

This problem is even more aggravated in follow-up studies between PET/CT and PET/MR based on this SUV-underestimation. A technical compensation for this issue might be that both available PET-components in simultaneous systems have a higher sensitivity, which might partially compensate for the diagnostic loss. However, there is currently no study available which investigates this systematically.

In those cases of incorrect underestimation, diffusion weighted imaging from the MR-component, for example, might be of help diagnostically. However, MR-sequences are usually even less standardized between different institutions than PET-systems.

### Summary of causes and magnitude of errors in SUV measurements

**Fig. 2:**
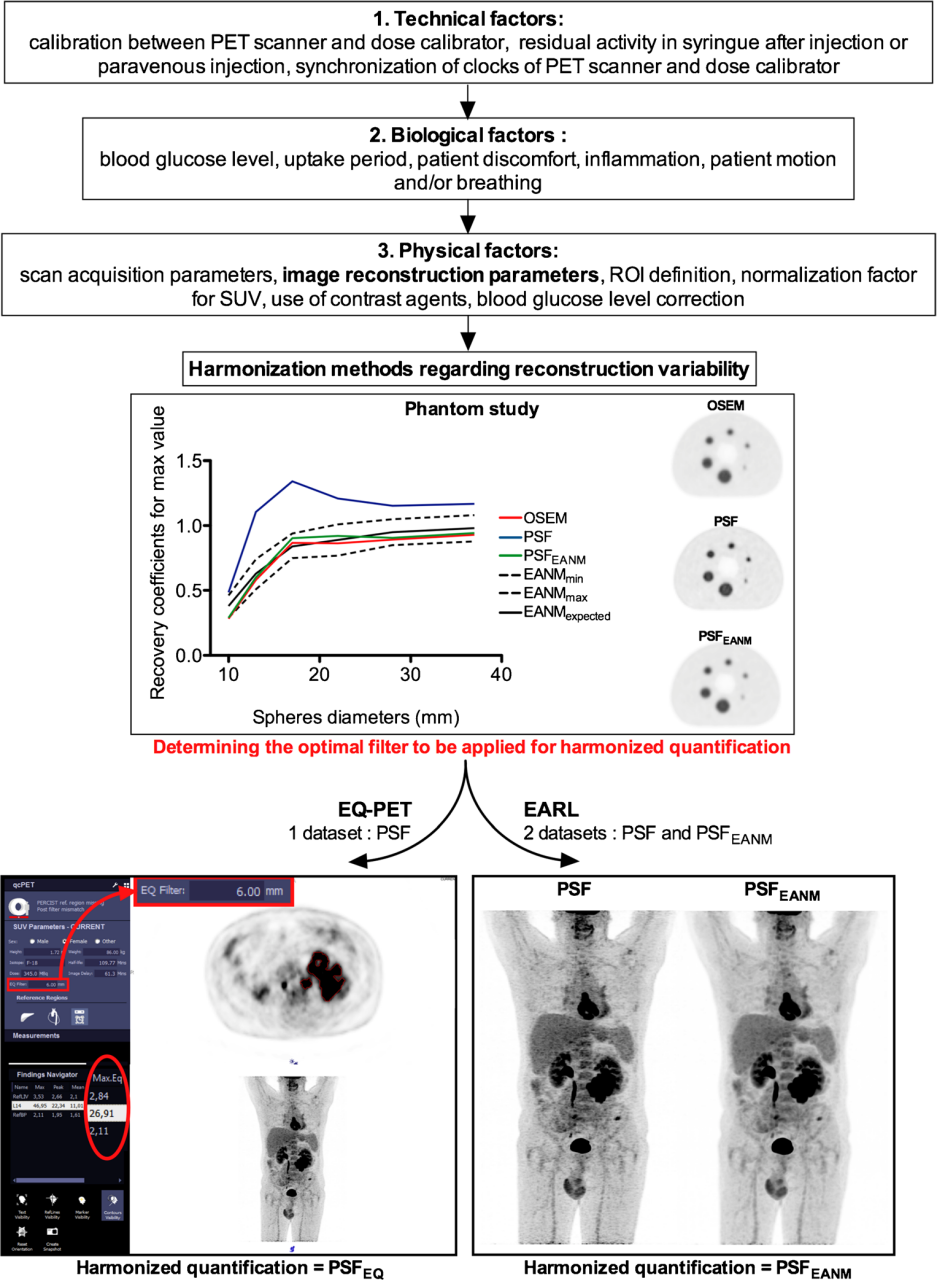
Illustration of reconstruction harmonization methods and brief summary of the main factors influencing SUV

The causes and the magnitudes of errors in SUV measurements have been described in detail elsewhere [28]. These errors can be classified into three categories and are briefly summarized in Fig. 2. It is worth mentioning that among the technical causes of errors in SUV calculation, reconstruction variability has taken a prominent place over the last decade, with technological improvements in PET technology having a huge impact on SUV measurements. For example, reconstructions including the PET/CT system resolution model (so-called PSF reconstruction), with no post-filtering, have been reported to increase SUV_max_ beyond 66% in small nodal metastases in breast cancer [29], or for NSCLC as reported by Kuhnert et al. The increase in PET quantitative metrics due to this algorithm will depend on the post filtering settings, but PSF reconstructions are usually used with little to no filtering. More recently, Bayesian penalized likelihood (BPL) reconstruction has been shown to improve tumor detection and to increase SUV metrics [30, 31]. A review of recent advancements in PET technology can be found elsewhere in this supplement [32].

### The issue of reconstruction variability among PET centers

In an international survey, Beyer et al. [33] reported that 52% of sites used alternative protocols with adapted reconstruction parameters. Of note, there is a reconstruction variability even between centers running similar systems: Sunderland et al. [34], from the SNMMI clinical trials network, reported that site-specific reconstruction parameters increased the quantitative variability among similar scanners, with post-reconstruction smoothing filters being the most influential parameter. In their survey involving 237 PET/CT systems in 170 international imaging centers, with technology advancements spanning more than a decade and covering the three major PET manufacturers (GE Healthcare, Siemens and Phillips Healthcare made up approximately 56%, 34% and 10%), more than 100 reconstruction parameters were reported. Rausch et al. [35] reported an overview of clinical PET/CT operations in Austria in a survey involving 12 PET centers (GE Healthcare, Phillips Healthcare and Siemens Healthcare made up 4/12, 7/12 and 2/12, respectively). Graham et al. [36] reported a survey in 15 US centers. Table 1 summarizes data available from these surveys. As can be seen in Table 1, all these reports suggest a huge variability in state of the art PET/CT system performance in the absence of a careful PET/CT system harmonization program.

**Table 1:** Summary of international and US surveys on PET/CT operation

ref	Centers/PET systems	Weight-based FDG injection	Fasting Period (h)	Injected activity MBq/Kg^*^	Uptake time (min)^*^	Acquisition time per bed position	Reconstruction parameters			
							matrix	iterations	subsets	Post-filtering Kernel (mm)
Rausch [35]	12/12	10/12	7.6 (4–12)	3.2–5	55 (45–75)	1min15s-3 min	128^2^–256^2^	2–4	18–32	0–6.4
Sunderland [34]	170/237	n/r	n/r	n/r	n/r	n/r	n/r	n/r	n/r	2–10
Graham [36]	15/n/a	3/15	>4	5.2–8.1	^**^(45–90)	2–7 min	n/a	n/a	n/a	n/a

n/a: not available

n/r: not relevant (phantom only studies)

^*^ Data are presented as mean (range)

^**^ Average value not reported

## Harmonization strategies

### From preparation of patient in the PET unit to acquisition and reconstruction

#### (EARL, UPICT)

A detailed review of various factors affecting SUV (and MATV, TLG) can be found in [28, 37, 38]. When a patient undergoes a PET/CT examination, errors may occur during the entire process of the study. During this process several steps can be identified, such as: (1) patient instruction, at least one day prior to the examination to ensure, e.g., that patient has fasted properly; (2) patient preparation and FDG administration; (3) PET/CT examination; (4) Image reconstruction/generation; (5) Image analysis and interpretation. A detailed overview of the various steps is summarized in the UPICT protocol and EANM version 2.0 guidelines [4, 39]. In all steps of the examination it is essential to mitigate the sources of errors [28]. From an image acquisition and reconstruction point, it is important to ensure that the PET/CT examination is of sufficient quality. The latter depends on (the combination of) patient weight, scan duration, FDG activity administered, PET/CT system sensitivity and image reconstruction methods and settings. To ensure sufficient image quality and harmonized image quantification, the EANM guideline gives specific recommendations for the (minimal) FDG activity to be administered in relation to patient weight and image acquisition parameters. Moreover, based on this guideline a PET/CT quality control program was launched in 2010 aiming at harmonizing image quality and quantification across sites and PET/CT systems. For SUV bias and recovery coefficients, EARL accreditation acceptance limits were established based on the results of a feasibility study performed on PET/CT systems currently used in clinical practice, including different types from different vendors. The specific aim of this EARL accreditation program is to ensure exchangeability or pooling of quantitative results in a multicenter setting, although the authors suggested that it is also beneficial to derive interpretation criteria for routine clinical use of quantitative PET/CT metrics.

The EARL program uses a specific set of quality control (QC) experiments. The first one aims to verify the basic calibration of the PET/CT relative to the dose calibrator used to measure the patient FDG activities. The experiment uses a simple uniform phantom; it is designed to ensure consistent calibrations between these two devices and thereby correct SUV calculations. This QC is required by EARL quarterly to verify that the accurate calibration of the accredited PET/CT system is ensured over time on site. The second QC requires the NEMA NU 2 image quality phantom and is used to derive the reconstruction settings that results in comparable SUVs across systems by harmonizing SUV recoveries. The EARL program provides harmonizing specifications for SUV recoveries, i.e. both lower and upper limits are provided, thereby aiming at minimizing differences in quantitative reads between sites, systems and reconstruction methods. This second QC is repeated annually and/or after major repairs of the PET/CT system.

The EARL accredited department pledges itself to perform all FDG PET/CT oncology examinations, at least all quantitative ones, strictly as described in the EANM guideline (updated version), to provide a minimum standard for the acquisition and interpretation of PET/CT scans, using the EARL approved parameters.

While most of the causes of errors in PET quantitative measurements can be overcome by complying with existing guidelines, from preparation of the patients to acquisition, a specific issue is related to reconstruction-dependent variations encountered with recently introduced advanced image reconstruction algorithms, such as those incorporating the point spread function (PSF) [40], or BPL reconstruction [31]. These new image reconstruction schemes have been shown to produce SUV metrics significantly higher than conventional ordered subset expectation maximization (OSEM) algorithms [29]. Consequently, an additional filtering step has to be used in order to meet harmonizing standards [4, 41, 42]. In this way the benefits of PSF reconstruction for visual interpretation can be combined with compliance to international quantitative harmonizing standards, as will be discussed below.

### Clinical validation of the EARL harmonization strategy

Given that centers running PET systems with advanced reconstruction algorithms are often willing to use them as such in order to achieve optimal tumor detection, EARL-accredited centers tend to use two PET datasets: one for optimal lesion detection and image interpretation, and a second (possibly filtered) one for harmonized quantification [41]. This strategy has been validated in several studies that mimicked a situation in which a patient would undergo pre- and post-therapy PET scans on different generation PET systems by comparing SUVs for an OSEM reconstruction known to meet the EANM harmonizing standards to a PSF or PSF + TOF reconstruction optimized for diagnostic purposes and then SUVs for a PSF or PSF + TOF EARL-compliant reconstruction.

In a series of 52 NSCLC with 195 lesions [41], Bland-Altman analysis demonstrated that the mean ratio between PSF_all pass_ and OSEM data was 1.48 (95% CI 1.06–1.91) and 1.37 (95% CI 0.89–1.85) for SUV_max_ and SUV_mean_, respectively. After having applied the appropriate filter, the mean ratios between PSF_EARL_ and OSEM data were 1.03 (95% CI 0.94–1.12) and 1.02 (95% CI 0.90–1.14) for SUV_max_ and SUV_mean_, respectively. Since no confounding factors (tumor size, intensity, and location) were found, this methodology could be used in any type of solid tumors.

### Second reconstruction versus software technology

To avoid the reconstruction of two datasets, a proprietary software solution, marketed as EQ.PET (Siemens, Oxford, UK), has been developed to simultaneously allow optimal lesion detection and harmonized quantification from a single dataset [42, 43]. This software simultaneously presents the reconstruction that provides optimal lesion detection for diagnostic interpretation with harmonized SUV results. EQ.PET is a patented automatic software system working “behind the scenes” without possibility for the imaging specialist to check the adequacy of region of interest placement. Both EARL harmonization strategy and EQ.PET software operations are illustrated in Fig. 2.

EQ PET has been validated in a series of 517 patients with NSCLC, non-Hodgkin lymphoma and metastatic melanomas [44]. In this prospective multicentre study, 1380 tumor lesions were studied and Bland-Altman analysis showed a mean ratio between PSF or PSF + TOF and OSEM of 1.46 (95%CI: 0.86–2.06) and 1.23 (95%CI: 0.95–1.51) for SUV_max_ and SUV_peak_, respectively. Application of the harmonizing software improved these ratios to 1.02 (95%CI: 0.88–1.16) and 1.04 (95%CI: 0.92–1.17) for SUV_max_ and SUV_peak_, respectively. It is noteworthy that in this study, two centers used similar PET equipment but different reconstruction parameters: one used PSF modeling and no post filtering, while the other used Gaussian filtering with a kernel depending on the patients’ body habitus. This well reflects the issue of reconstruction variability pointed out by several European and US surveys and described above.

Lasnon et al. [45] compared the EQ.PET methodology (PSF_EQ_) with the use of a second harmonized reconstruction (PSF_EARL_) in a series of 55 NSCLC cancer patients (171 lesions) imaged on a system equipped with PSF modeling and showed that the mean PSF_EARL_/PSF_EQ_ ratio for SUV_max_ and SUV_peak_ were 1.01 (95%CI: 0.96–1.06) and 1.01 (95%CI: 0.97–1.04), respectively.

Therefore reconstruction-dependency in SUVs can be overcome by using two reconstructions for harmonized quantification, and optimal diagnosis and could be managed by using software approaches like the EQ.PET technology, provided it is widely available and vendor neutral. Both technologies produce similar results, the software solution sparing reconstruction and interpretation time.

### Harmonization and liver-based scales

The Deauville score (DS) compares FDG uptake in the residual masses with that in the mediastinal blood pool and in the liver, following chemotherapy in Hodgkin lymphomas (HL) and non-Hodgkin lymphomas (NHL) [46]. DS is widely used from interim and end-treatment PET. In order to better characterize non-responding disease (i.e uptake slightly superior or greatly superior to liver background, defined as DS 4 and DS 5, respectively), it has been suggested to compute lesion/liver ratio and to use a 1.3 cutoff value.

Based on the SUV formulae described above, one could assume that the use of a ratio would allow one to remove the reconstruction variability, the hypothesis being that an overestimation due to the use of an advanced reconstruction algorithm would equally impact the lesion and the liver SUVs. In a series of 23 NHL patients with a total of 388 lesions [47], PSF reconstruction was shown to increase the tumor-to-liver ratio by 31% (ratio 1.31, 95% CI: 0.79–1.82) compared to the conventional OSEM algorithm. After having applied a Gaussian filter chosen to meet the EANM harmonizing standards (PSF_EARL_), the ratio of the tumor- to-liver ratio for PSF_EARL_ and OSEM was found to be 1.06 (95% CI:0.93–1.18), with a narrow 95% confidence interval. Therefore, the lesion/liver ratio, if used as a discriminator between a positive and negative exam in NHL patients, is PET system and image reconstruction method dependent, and harmonization is thus still warranted. This is in line with a study from Kuhnert et al. [48], in which SUVs were compared in PSF + TOF reconstruction versus OSEM in a series of 40 lung cancer patients. Their study demonstrated that SUVs were constantly increased in PSF + TOF images, despite normalization to the liver. On average, the observed increase was 60% and 30% for SUV_max_ and SUV_peak_, respectively. These values can be compared to those observed by Lasnon et al. [41] using PSF modeling with no filtering and described in detail above.

Taken together, these data show that harmonization is warranted not only for SUV metrics, but also for tumor/liver ratios, which is of importance in the context of ongoing efforts to better stratify lymphoma patients with persistent disease, as discussed during the recent Menton congresses on Lymphoma and pointed out in the review by Barrington et al. [49].

### Harmonization and therapy assessment with EORTC response criteria and PERCIST

Various schema based on the degree of SUV change after treatment have been proposed in an effort to bring consistency to the classification of responses across trials, emulating the use of the RECIST for CT. A 25% threshold in SUV_max_ variation and a 30% variation in SUV_peak_ are used to discriminate between responding and non-responding tumors [50]. The EORTC criteria and PERCIST can be used not only for trials but also in daily routine.

**Fig. 3:**
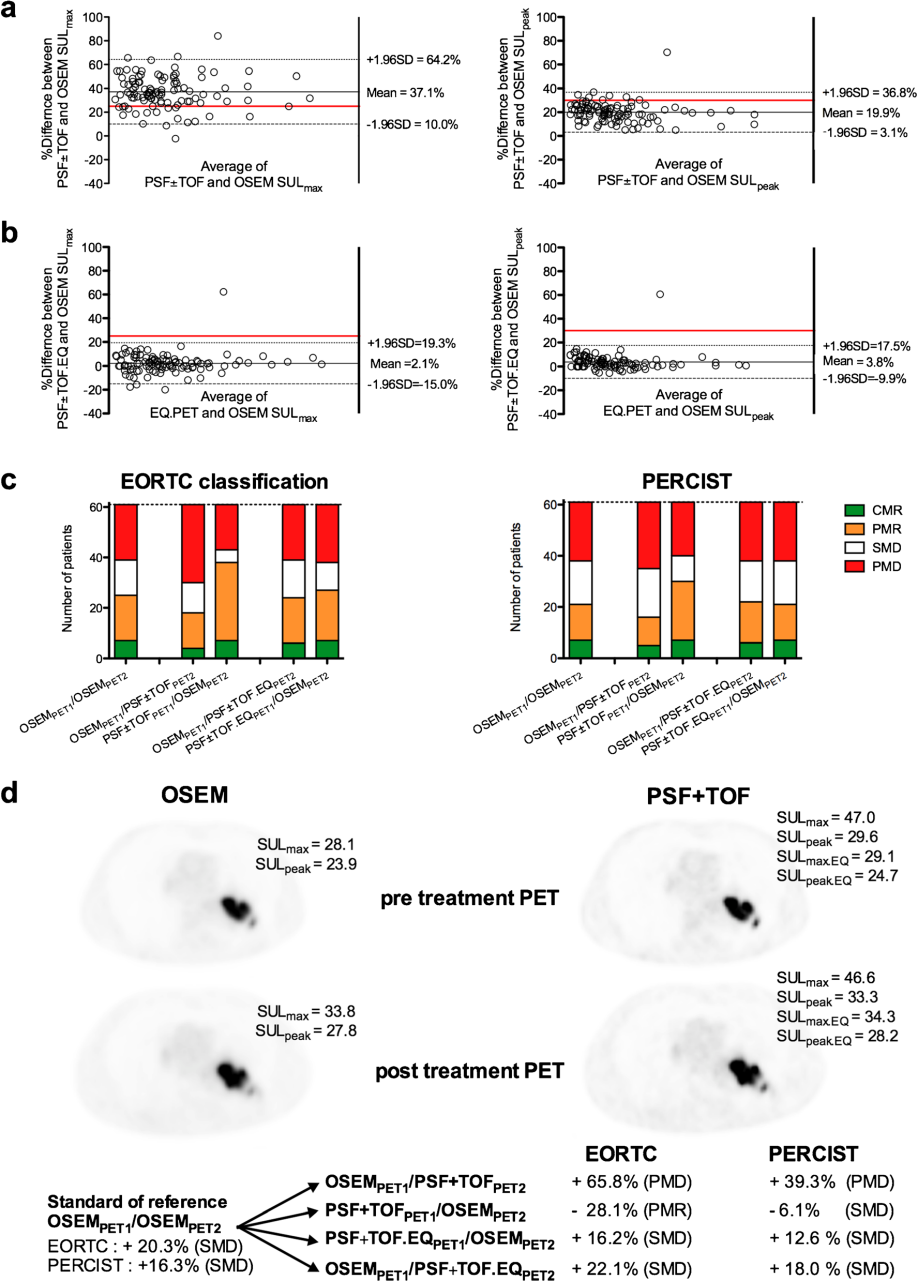
Effect of reconstruction inconsistencies and impact of harmonization on therapy assessment with EORTC response criteria and PERCIST. Relationship between standardized uptake values normalized to lean body mass (SUL)_max_ and SUL_peak_ in lesions extracted from PSF ± TOF (**a**) or PSF ± TOF.EQ (**b**) and OSEM images, assessed using Bland-Altman plots. Of note is the greater sensitivity of SUV_max_ to reconstruction variability, compared to SUV_peak_: the number of cases exceeding the threshold to discriminate between SMD and PMD, due to reconstruction inconsistency, is higher for SUV_max_. Conversely, PERCIST appears less sensitive than EORTC criteria to reconstruction inconsistency between pre- and post-treatment scans: panel **c** displays EORTC classification and PERCIST for the standard of reference (OSEM_PET1_/OSEM_PET2_) and for other scenarios. **d**: representative images of a 72-year-old male patient with NSCLC treated by chemotherapy, classified as SMD according to the standard of reference. The use of OSEM for baseline scan and PSF + TOF for post-treatment scan, mimicking a system upgrade during a trial, would lead to PMD classification for both EORTC and PERCIST, while the use of harmonized data would correctly classify the patient

As shown in Fig.3, reconstruction variability can lead to overestimation of SUV_max_ and SUV_peak_, exceeding the thresholds used to discriminate between responding (partial metabolic response) and non-responding (stable or progressive metabolic disease) patients. Also noticeable is the greater sensitivity of SUV_max_ to reconstruction variability, compared to SUV_peak_. Conversely, one could expect PERCIST to be less sensitive than EORTC criteria to reconstruction inconsistencies between pre- and post-treatment scans.

The impact of reconstruction inconsistency on therapy assessment was investigated in two studies: a prospective multicentre study involving 86 patients with NSCLC, colorectal liver metastases and melanoma metastases focused on PERCIST [51], and a single-centre series of 61 NSCLC specifically addressing the issue of the relative sensitivity of EORCT criteria and PERCIST to reconstruction variability [52]. In both studies, the use of a conventional OSEM algorithm for the pre- and post-treatment scans was used as the standard of reference (OSEM_PET1_/OSEM_PET2_ scenario).

For the OSEM_PET1_/OSEM_PET2_ scenario, the change in SUL_peak_ was −63.9 ± 22.4 and +60.7 ± 19.7 in the groups of tumors showing a decrease and an increase in FDG uptake, respectively, while the change in SUL_max_ was −57.5 ± 23.4 and +63.4 ± 26.4 in the groups of tumors showing a decrease and an increase in 18F–FDG uptake, respectively. The use of PSF or PSF + TOF reconstruction affected tumor classication, depending on whether this reconstruction was used for the pre- or post-treatment scans. For example, taking the OSEM_PET1_/PSF or PSF + TOF_PET2_ scenario (a situation that would be faced if a system upgrade were done during a trial), would decrease the apparent reduction in responding tumors and would increase the percentage change in progressing tumors. Conversely, this was shown to affect both the EORTC and PERCIST classifications. In agreement with the higher reconstruction-dependency of SUV_max_ compared to SUV_peak_, the discordances between scenarios involving reconstruction inconsistencies and the standard of reference (OSEM_PET1_/OSEM_PET2_ scenario) were more frequent for SUV_max_/EORTC. Of note, the potential impact of these discordances was more important for the EORTC compared to PERCIST, more patients’ classifications being changed from responder [partial metabolic response (PMR) or complete metabolic response (CMR)] to non-responder [stable metabolic disease (SMD) or progressive metabolic disease ( PMD)]. After having applied an appropriate filter to comply with the EANM harmonizing standards, agreement levels between the OSEM_PET1_/OSEM_PET2_ scenario and other scenarios involving reconstruction inconsistency were found to be almost perfect, with narrow confidence intervals. Figure 3 displays the percentage changes for the different scenarios and PERCIST or EORTC classifications.

Of note, PERCIST recommend using the lesion harboring the highest FDG uptake as a target lesion and do not require the same target lesion to be used on pre- and post-treatment scans. In that setting, given that new reconstruction algorithms have been shown to improve lesion detectability, a different target lesion could be chosen on OSEM and PSF images. In the study from Quak et al. [52], a change in selected PERCIST target lesion occurred in only 3 of 172 scans (2%). Also, among patients classified as PMD because of the appearance of new lesions, OSEM and PSF or PSF + TOF performed equally in detecting these new lesions, despite the potential for PSF reconstruction to detect smaller cancer lesions compared with OSEM reconstruction.

### Harmonization and MATV

Because two MATVs of a given tumor could, in theory, not be identical, i.e. representing different metabolic parts of the tumor, validation of the EARL harmonization strategy requires that MATV are compared not only in terms of absolute and relative values, but also using a representative geometrical description of MATV changes, combining volume and positional changes. In that setting, Dice’s and concordance indices are frequently used. Their values vary between 0 if the MATVs are completely disjointed and 1 if the MATVs match perfectly in terms of size, shape and location.

Using the 40% isocontour method and taking MATV delineated on OSEM images as a reference standard, Lasnon et al. [53] showed in 18 NSCLC patients that the use of EARL-compliant images led to significantly higher Dice’s coefficients (median value = 0.96 vs 0.77, *P* < 0.0001) and concordances indices (median value = 0.92 vs 0.64, *P* < 0.0001), compared to the use of PSF images optimized of diagnostic. This shows that automatically contouring tumors on EARL-compliant PSF images with the widely adopted automatic isocontour methodology is an accurate means of getting rid of reconstruction variability in MATV delineation.

### Using PET EARL-compliant images to evaluate tumor heterogeneity

Heterogeneity metrics are emergent and alternative PET measurements [54–57]. The most promising approach for heterogeneity quantification is textural features (TF) analysis. Recently, the impact of reconstructions on TF values has been highlighted and the efficacy of harmonization programs initially developed for standard SUV metrics has been tested: in a series of 60 NSCLC patients, several ^18^F–FDG heterogeneity metrics were compared in PSF, PSF-filtered (EARL-compliant) and OSEM reconstructed images. Tested TF were CH_AUC_ (first-order metric); entropy, dissimilarity and correlation (second-order metrics); ZP and HILAE (third-order metrics).

When using the same volume of interest (VOI) on the three reconstructions (thus avoiding a VOI-related bias), Lasnon et al. [58] found significant differences between OSEM and PSF images for all heterogeneity metrics except for entropy and ZP; the latter could therefore be used in the case of multicentre studies within centers using different reconstruction settings. When comparing heterogeneity metrics extracted from OSEM and PSF_7_ images, none exhibited significant differences, emphasizing that the quantifiable heterogeneity contents of PSF_7_ images are very close to those in OSEM images whatever the MATV considered, and supporting the use of harmonization strategies in multicentre studies using TF as biomarkers. However, it is noteworthy that overall, PSF images displayed higher heterogeneity and higher ranges of heterogeneity, especially when analyzing the largest tumors (>1cm^3^). This suggests that PSF-reconstructed images could be more accurate in discriminating different levels of intra-tumoural heterogeneity than OSEM-reconstructed images, and that when available, PSF-images should be exploited in addition to EARL-compliant images.

### Implementing the EARL strategy in daily practice and multicentre studies: Results from the EARL electronic survey (Fig. 4)

An electronic survey took place over a two-week period in September 2016 among EARL-accredited centers. At the time of this survey, 169 centers were accredited. The link to this online survey was sent to the referring physician or physicist of each centre. One reminder was sent 48H before the closure of the survey; 115 centers viewed the questionnaire and 51 centers responded, meaning a response rate of 44%.

**Fig. 4:**
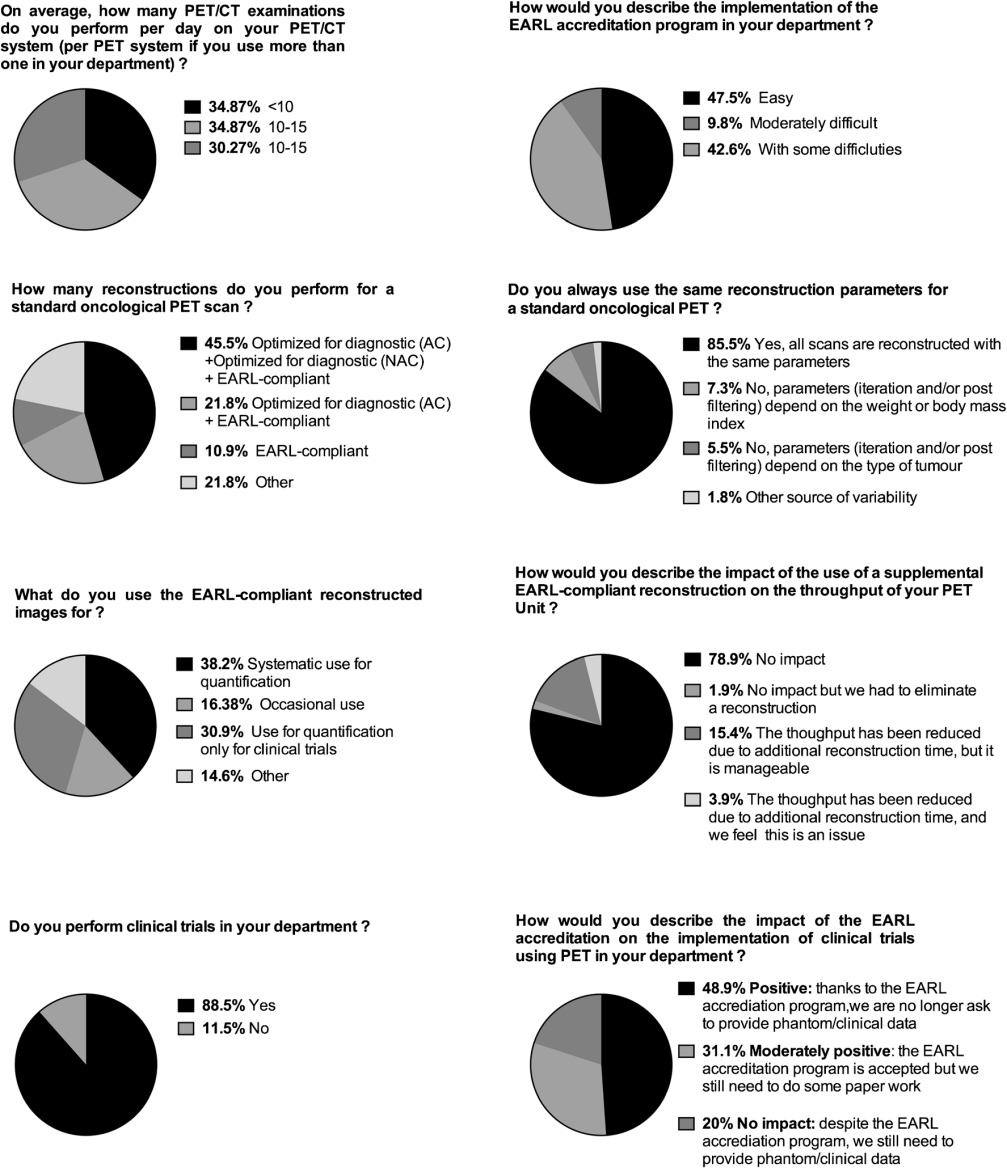
Results from the EARL electronic survey. Data are displayed as pie charts

Most of the centers that responded to the survey are centers performing more than 15 PET examinations per day and participating in clinical trials. Half of these centers reported the implementation of the EARL accreditation program as easy.

With regards to daily practice, most of the centers use a reconstruction optimized for diagnostic images in addition to the use of EARL compliant images, half of them using three reconstructions for a standard oncological PET scan (i.e. images optimized for diagnostic, corrected and uncorrected for attenuation + EARL-compliant images, the latter being systematically used for quantification in 38% of centers and only for clinical trials in a third of the centers). Given the increasing number of PET centers running more than one PET system, the systematic use of EARL images is likely to increase, as always scanning a patient on the same PET scanner is difficult.

In line with the number of reconstructions being used in EARL-accredited centers, most of the centers reported the lack of impact of the EARL program on the throughput of their unit. When it comes to clinical trials, the impact of the EARL program was judged positive in half of the cases, but a third of the centers reported that paperwork is still needed.

## Future evolutions and imaging guideline updates

### Weight measurement: A neglected cause of variability?

In a survey involving 513 consecutive patients in an EARL-accredited centre, Lasnon et al. [59] showed that, compared to the actual weight, using weight reported on the PET request forms led to an overestimation and an underestimation greater than 10% in 35 (7.4%) and 23 (4.9%) patients, respectively. Based on the SUV formulae, an overestimation of patient’s weight can lead to an overestimation of SUV metrics, and vice versa*.* These errors may hamper efforts to meet quantitative harmonizing standards. Based on this survey, two strategies can be proposed: either to systematically ask patients to weigh themselves 48 h before the PET examination when they are called-up, or, especially in other PET units where patients are not systematically called-up, to weigh patients upon their arrival in the PET unit on a calibrated weighing scale. This last option could be easily generalized to all patients, (i.e. not only those imaged within clinical trials, as suggested by the UPICT protocol [39] but also those being scanned in clinical routine).

### Lean body mass (LBM) versus weight for SUV calculation: How to evaluate LBM

PERCIST [60] recommend the use of SUV normalized by lean body mass (SUV_LBM_) rather than SUV normalized by body weight (SUV_BW_). Indeed, SUV_LBM_ has been shown to be more consistent by taking into account that adipose tissue, the amount of which is highly variable among patients, does not significantly accumulate FDG. Regarding SUV definition, this theoretically leads to an underestimation of SUV_BW_ in obese patients. There are two main methods of LBM calculation: indirect estimation by predictive equations (PEs) and direct determination by using computed tomography (CT).

Modern PET/CT systems use PEs based on basic anthropometric parameters (gender, body weight, height ± age). For example, one of the most common, called the James equation, is defined as follows:

$$ \begin{array}{l}{LBM}_{\mathrm{James}}=1.1\times BW-128\times {\left(\frac{BW}{\mathrm{Height}}\right)}^2\kern0.5em  for\kern0.5em  men\\ {}{LBM}_{\mathrm{James}}=1.07\times BW-148\times {\left(\frac{BW}{\mathrm{Height}}\right)}^2\kern0.5em  for\kern0.5em  women\end{array} $$

However, these equations have some limitations that hamper their reliability. It has been shown that most of the PEs were significantly different from LBM derived from dual-energy x-ray absorptiometry, which is one of the most accurate reference methods, with wide variations in LBM estimation [61]. It is noticeable that this study included some PEs previously used to normalize SUV. Moreover, Tahari et al. demonstrated inappropriately low hepatic level SUL values in female and male obese patients when using the James equation described above [3]. Therefore, instead of estimation, an individual LBM measurement seems to be more reliable.

As all patients now have a systematic CT scan coupled with their PET acquisitions, some have proposed using this source of information to directly determine LBM based on Hounsfield densities. The fat peak is well defined on CT histogram (from −190 to −30 HU) and depends little on the image noise, so no CT parameter adaption is required [62]. For the great majority of patients, the field of view (FOV) covers only skull to mid-thighs, but several studies have demonstrated that the estimation of LBM on a limited FOV has an excellent agreement with the LBM measured on a whole-body CT [1]. When comparing PEs and CT LBM determinations, substantial errors were found between SUL calculated with PEs compared to CT, with errors in individual SUL values ranging from 25% to 51% [63].

Obesity being a progressing disease, SUL determination improvements must be a matter of major concern, as it is an important endpoint in the outcome of oncologic patients.

## New harmonization initiatives

### New isotopes

The current EARL program was developed to harmonize PET/CT system performance for multicenter FDG PET/CT studies. Although the focus was on FDG and quality control experiments for obtaining accreditation use ^18^F(FDG) as a radioisotope, the program is applicable to any other ^18^F labeled radiopharmaceutical. New EARL initiatives are underway to address the use of other radioisotopes, such as ^89^Zr [64] and ^68^Ga. In most cases the EARL approved acquisition and reconstruction parameters (for FDG) may be applied directly to obtain harmonized PET/CT performance for these other isotopes. However, when using isotopes other than ^18^F, several isotope dependent issues need to be considered. First of all, the positron range may be substantially longer than that of ^18^F, which is, in particular, the case for both ^89^Zr and ^68^Ga. The longer positron range results in lower SUV or contrast recoveries for smaller objects (<1.5 cm diameter). Yet, the effects of positron range on observed contrast recovery should be the same, regardless PET/CT systems used. A pragmatic approach for harmonizing PET/CT systems for ^89^Zr and ^68^Ga would be to simply use the ^18^F(FDG) approved settings, thereby avoiding the need to install multiple isotope specific EARL protocols on the PET/CT system, and to validate only ^89^Zr and ^68^Ga recoveries under these conditions. Secondly, a proper cross-validation of PET/CT calibration with that of the dose calibrator used to determine the patient activities is still warranted. The latter is sometimes hampered by the lack of the appropriate isotope information on either the PET/CT system or dose calibrator. Use of incorrect isotope settings will result in incorrect decay correction and use of the wrong positron abundance. Both issues will result in incorrect measurement of the activity concentrations or activities by the systems, which is unacceptable for clinical use. Therefore, EARL will set up these new programs in order to facilitate the use of these potentially interesting and widely used new isotopes in multicenter studies.

### New PET technologies

Of importance to note is that EARL is a multicenter standard aiming at harmonizing PET/CT systems regardless of their technological capabilities. The standards were set to achieve the highest common denominator for state of the art PET/CT systems. PET-only systems were not used to derive the standards and the standards were not defined by the worst performing systems. Yet, given the recent developments in PET technologies, such as the introduction of PSF reconstructions and digital PET detectors, the EARL standard may need to be updated. It should be noted, however, that a substantial fraction of the PET/CT systems in Europe still does not have PSF reconstruction capabilities, let alone digital PET detectors. Update of EARL is inevitable, but its implementation depends on the installed base of PET/CT systems in Europe and the support of vendors to accommodate new EARL standards. At present, efforts supported by EARL and the Quantitative Imaging Biomarkers Alliance (QIBA) [65] are undertaking to obtain a new set of experiments to test the feasibility of harmonizing PET/CT systems with PSF reconstructions, possibly in combination with use of SUV_peak_, and even digital PET detectors, but data are still preliminary. Once a new standard has been implemented its impact on quantitative PET results and (quantitative) PET interpretations should be addressed. It can be expected that by using a standard that facilitates the use of new PET technologies, SUVs will be higher and MATVs smaller. The translation of interpretation criteria from an old to a new standard could be addressed either by performing multiple reconstructions or by use of a post reconstruction filter, i.e. the same strategies currently followed by most sites to obtain images optimized for visual interpretation and for multicenter quantification. Although the latter is a challenge, the transition from one standard to another is more preferable than the use of quantitative PET in an unstandardized chaotic manner, as the surveys of Sunderland et al. and Graham et al. have revealed [34, 36].

### Harmonization for PET/MR devices

Combined or integrated PET/MR was introduced several years ago and has gained increased interest, although mainly in the academic world, in exploring its capabilities and use. In most PET/MR systems the PET component performs similarly to its PET/CT counterparts, although some lack the use of time of flight, while other systems already use digital PET technologies. Despite these technical differences, the approach to harmonizing the PET performance is not different from that of PET/CT systems. A particular challenge for PET/MR is the lack of PET phantoms that are commonly used for the calibration and quality control of PET/CT systems. But Boellaard et al. [24] recently showed that all PET/MR systems have implemented protocols and image reconstruction methods that allow the use of uniform cylinders to calibrate the PET(/MR) system as well as the use of the NEMA Image Quality phantom to perform NEMA and/or EARL Image Quality QC experiments. In this way the current EARL accreditation program for PET/CT can be applied PET/MR systems as well. Although the latter assures harmonized performance of the PET component of the PET/MR from a physics or technical perspective, quantification in humans may still be hampered by limitations in the commercially provided solutions for MR based attenuation correction. An overview of the various issues related to quantitative PET/MR imaging can be found in [66]. Moreover, it has also been shown that the commercially provided MR based attenuation correction methods may suffer from poor repeatability and reproducibility (between systems) as shown by Beyer et al. [23]. Yet, as discussed earlier, more advanced and accurate MR based attenuation correction methods have been developed; when these new methods are employed the quantitative accuracy of PET/MR will be equivalent to that of PET/CT for most cases, but validation and inspection of the attenuation correction maps remains warranted.

## Conclusions and perspectives

Use of quantitative PET/CT parameters, such as SUVs or MATVs, as imaging biomarkers in multicentre trials or in sites equipped with multiple scanners requires that these parameters be comparable among patients, regardless of the PET/CT system used. The EANM/EARL program, one of the international harmonization programs aiming at using FDG PET as a quantitative imaging biomarker in clinical trials, requires a specific set of quality control experiments, including a set of PET images with NEMA NU-2 anthropomorphic phantom-based filtering to harmonize SUVs to the EANM standards. EARL-accredited centers tend to use two PET datasets: one for optimal lesion detection and image interpretation, and a filtered one for harmonized quantification. In this way the benefits of advanced reconstruction algorithms such as PSF or PSF + TOF for visual interpretation can be combined with compliance to international quantitative harmonizing standards. The EARL accreditation program has been proven to be effective in getting harmonized quantitative values, in particular by overcoming algorithm and reconstruction variability across PET systems. Its clinical validation was made in a wide range of tumor types, not only for SUV metrics, but also for MATV and heterogeneity features. The need for harmonization in therapy assessment and the efficiency of the EARL program in this setting have been demonstrated for both the EORTC response criteria and PERCIST. A recent survey across EARL accredited sites suggests that EARL accreditation and use of EARL accredited protocols, either by themselves or in combination with locally preferred settings optimized for lesion detection, do not hamper clinical routine and throughput.

## References

[1] Decazes P, Metivier D, Rouquette A, Talbot JN, Kerrou K (2016). A method to improve the semiquantification of 18F-FDG uptake: reliability of the estimated lean body mass using the conventional, low-dose CT from PET/CT. J Nucl med.

[2] Devriese J, Beels L, Maes A, Van De Wiele C, Gheysens O, Pottel H. Review of clinically accessible methods to determine lean body mass for normalization of standardized uptake values. The quarterly journal of nuclear medicine and molecular imaging: official publication of the Italian Association of Nuclear Medicine (AIMN) and the International Association of Radiopharmacology (IAR), and Section of the So. 2016;60:1–11. 26576735

[3] Tahari AK, Chien D, Azadi JR, Wahl RL (2014). Optimum lean body formulation for correction of standardized uptake value in PET imaging. J Nucl med.

[4] Boellaard R, Delgado-Bolton R, Oyen WJ, Giammarile F, Tatsch K, Eschner W, et al. FDG PET/CT: EANM procedure guidelines for tumour imaging: version 2.0. Eur J Nucl Med Mol Imaging. 2015;42:328–54. doi:10.1007/s00259-014-2961-x. 10.1007/s00259-014-2961-x PMC4315529 25452219

[5] Boellaard R, O’Doherty MJ, Weber WA, Mottaghy FM, Lonsdale MN, Stroobants SG, et al. FDG PET and PET/CT: EANM procedure guidelines for tumour PET imaging: version 1.0. Eur J Nucl Med Mol Imaging. 2010;37:181–200. doi:10.1007/s00259-009-1297-4. 10.1007/s00259-009-1297-4 PMC2791475 19915839

[6] van Baardwijk A, Bosmans G, Boersma L, Buijsen J, Wanders S, Hochstenbag M (2007). PET-CT-based auto-contouring in non-small-cell lung cancer correlates with pathology and reduces interobserver variability in the delineation of the primary tumor and involved nodal volumes. Int J Radiat Oncol Biol Phys.

[7] Cheebsumon P, Boellaard R, de Ruysscher D, van Elmpt W, van Baardwijk A, Yaqub M (2012). Assessment of tumour size in PET/CT lung cancer studies: PET- and CT-based methods compared to pathology. EJNMMI res.

[8] Cheebsumon P, van Velden FH, Yaqub M, Frings V, de Langen AJ, Hoekstra OS (2011). Effects of image characteristics on performance of tumor delineation methods: a test-retest assessment. Journal of Nuclear Medicine : Official Publication, Society of Nuclear Medicine.

[9] Cheebsumon P, Yaqub M, van Velden FH, Hoekstra OS, Lammertsma AA, Boellaard R (2011). Impact of [(1)(8)F]FDG PET imaging parameters on automatic tumour delineation: need for improved tumour delineation methodology. Eur J Nucl Med Mol Imaging.

[10] Mikhaeel NG, Smith D, Dunn JT, Phillips M, Moller H, Fields PA (2016). Combination of baseline metabolic tumour volume and early response on PET/CT improves progression-free survival prediction in DLBCL. Eur J Nucl Med Mol Imaging.

[11] Pierce LA, Elston BF, Clunie DA, Nelson D, Kinahan PE (2015). A digital reference object to analyze calculation accuracy of PET standardized uptake value. Radiology.

[12] Kalemis A, Delattre BM, Heinzer S (2013). Sequential whole-body PET/MR scanner: concept, clinical use, and optimisation after two years in the clinic. The manufacturer’s perspective. Magma (New York, NY).

[13] Delso G, Furst S, Jakoby B, Ladebeck R, Ganter C, Nekolla SG (2011). Performance measurements of the Siemens mMR integrated whole-body PET/MR scanner. J Nucl med.

[14] Grant AM, Deller TW, Khalighi MM, Maramraju SH, Delso G, Levin CS (2016). NEMA NU 2-2012 performance studies for the SiPM-based ToF-PET component of the GE SIGNA PET/MR system. Med Phys.

[15] Delso G, Wiesinger F, Sacolick LI, Kaushik SS, Shanbhag DD, Hullner M (2015). Clinical evaluation of zero-echo-time MR imaging for the segmentation of the skull. J Nucl med.

[16] Wiesinger F, Sacolick LI, Menini A, Kaushik SS, Ahn S, Veit-Haibach P (2016). Zero TE MR bone imaging in the head. Magn Reson med.

[17] Burgos N, Cardoso MJ, Modat M, Punwani S, Atkinson D, Arridge SR (2015). CT synthesis in the head & neck region for PET/MR attenuation correction: an iterative multi-atlas approach. EJNMMI Physics..

[18] Burgos N, Cardoso MJ, Thielemans K, Modat M, Dickson J, Schott JM (2015). Multi-contrast attenuation map synthesis for PET/MR scanners: assessment on FDG and Florbetapir PET tracers. Eur J Nucl med Mol Imaging.

[19] Leynes AP, Yang J, Shanbhag DD, Kaushik SS, Seo Y, Hope TA (2017). Hybrid ZTE/Dixon MR-based attenuation correction for quantitative uptake estimation of pelvic lesions in PET/MRI. Med Phys.

[20] Sekine T, Buck A, Delso G, Ter Voert EE, Huellner M, Veit-Haibach P (2016). Evaluation of atlas-based attenuation correction for integrated PET/MR in human brain: application of a head atlas and comparison to true CT-based attenuation correction. J Nucl med.

[21] Yang J, Jian Y, Jenkins N, Behr SC, Hope TA, Larson PE, et al. Quantitative evaluation of atlas-based attenuation correction for brain PET in an integrated time-of-flight PET/MR imaging system. Radiology. 2017;161603 doi:10.1148/radiol.2017161603. 10.1148/radiol.2017161603 28234560

[22] Ziegler S, Jakoby BW, Braun H, Paulus DH, Quick HH (2015). NEMA image quality phantom measurements and attenuation correction in integrated PET/MR hybrid imaging. EJNMMI Physics.

[23] Beyer T, Lassen ML, Boellaard R, Delso G, Yaqub M, Sattler B (2016). Investigating the state-of-the-art in whole-body MR-based attenuation correction: an intra-individual, inter-system, inventory study on three clinical PET/MR systems. Magma (New York, NY).

[24] Boellaard R, Rausch I, Beyer T, Delso G, Yaqub M, Quick HH (2015). Quality control for quantitative multicenter whole-body PET/MR studies: a NEMA image quality phantom study with three current PET/MR systems. Med Phys.

[25] Boellaard R, Hofman MB, Hoekstra OS, Lammertsma AA (2014). Accurate PET/MR quantification using time of flight MLAA image reconstruction. Molecular Imaging and Biology : MIB : the Official Publication of the Academy of Molecular Imaging..

[26] Nuyts J, Dupont P, Stroobants S, Benninck R, Mortelmans L, Suetens P (1999). Simultaneous maximum a posteriori reconstruction of attenuation and activity distributions from emission sinograms. IEEE Trans med Imaging.

[27] Samarin A, Burger C, Wollenweber SD, Crook DW, Burger IA, Schmid DT (2012). PET/MR imaging of bone lesions--implications for PET quantification from imperfect attenuation correction. Eur J Nucl Med Mol Imaging.

[28] Boellaard R (2009). Standards for PET image acquisition and quantitative data analysis. J Nucl Med.

[29] Bellevre D, Blanc Fournier C, Switsers O, Dugue AE, Levy C, Allouache D (2014). Staging the axilla in breast cancer patients with (1)(8)F-FDG PET: how small are the metastases that we can detect with new generation clinical PET systems?. Eur J Nucl Med Mol Imaging.

[30] Parvizi N, Franklin JM, McGowan DR, Teoh EJ, Bradley KM, Gleeson FV. Does a novel penalized likelihood reconstruction of 18F-FDG PET-CT improve signal-to-background in colorectal liver metastases? Eur J Radiol. 2015; doi:10.1016/j.ejrad.2015.06.025. 10.1016/j.ejrad.2015.06.025 26163992

[31] Teoh EJ, McGowan DR, Macpherson RE, Bradley KM, Gleeson FV. Phantom and clinical evaluation of the Bayesian penalized likelihood reconstruction algorithm Q.Clear on an LYSO PET/CT system. J Nucl Med. 2015; doi:10.2967/jnumed.115.159301. 10.2967/jnumed.115.159301 PMC4558942 26159585

[32] van der Vos CS, Koopman D, S. R, Arends AJ, Boellaard R, van Dalen JA, et al. Quantification, improvement and harmonization of small lesion detection with state-of-the-art PET. Eur J Nucl Med Mol Imaging. 2017; doi:10.1007/s00259-017-3727-z. 10.1007/s00259-017-3727-z PMC5541089 28687866

[33] Beyer T, Czernin J, Freudenberg LS (2011). Variations in clinical PET/CT operations: results of an international survey of active PET/CT users. J Nucl med.

[34] Sunderland JJ, Christian PE (2015). Quantitative PET/CT scanner performance characterization based upon the society of nuclear medicine and molecular imaging clinical trials network oncology clinical simulator phantom. J Nucl med.

[35] Rausch I, Bergmann H, Geist B, Schaffarich M, Hirtl A, Hacker M (2014). Variation of system performance, quality control standards and adherence to international FDG-PET/CT imaging guidelines. A national survey of PET/CT operations in Austria. Nuklearmedizin Nuclear Medicine.

[36] Graham MM, Badawi RD, Wahl RL (2011). Variations in PET/CT methodology for oncologic imaging at U.S. academic medical centers: an imaging response assessment team survey. J Nucl med.

[37] Boellaard R (2011). Methodological aspects of multicenter studies with quantitative PET. Methods Mol Biol.

[38] Boellaard R (2012). Mutatis mutandis: harmonize the standard!. J Nucl med.

[39] Graham MM, Wahl RL, Hoffman JM, Yap JT, Sunderland JJ, Boellaard R (2015). Summary of the UPICT protocol for 18F-FDG PET/CT imaging in oncology clinical trials. J Nucl med.

[40] Panin VY, Kehren F, Michel C, Casey M (2006). Fully 3-D PET reconstruction with system matrix derived from point source measurements. IEEE Trans med Imaging.

[41] Lasnon C, Desmonts C, Quak E, Gervais R, Do P, Dubos-Arvis C (2013). Harmonizing SUVs in multicentre trials when using different generation PET systems: prospective validation in non-small cell lung cancer patients. Eur J Nucl med Mol Imaging.

[42] Quak E, Le Roux PY, Hofman MS, Robin P, Bourhis D, Callahan J, et al. Harmonizing FDG PET quantification while maintaining optimal lesion detection: prospective multicentre validation in 517 oncology patients. Eur J Nucl med Mol Imaging. 2015; doi:10.1007/s00259-015-3128-0. 10.1007/s00259-015-3128-0 PMC4623085 26219870

[43] Kelly MD, Declerck JM (2011). SUVref: reducing reconstruction-dependent variation in PET SUV. EJNMMI res.

[44] Quak E, Le Roux PY, Hofman MS, Robin P, Bourhis D, Callahan J (2015). Harmonizing FDG PET quantification while maintaining optimal lesion detection: prospective multicentre validation in 517 oncology patients. Eur J Nucl med Mol Imaging.

[45] Lasnon C, Salomon T, Desmonts C, Do P, Oulkhouir Y, Madelaine J, et al. Generating harmonized SUV within the EANM EARL accreditation program: software approach versus EARL-compliant reconstruction. Ann Nucl med. 2016; doi:10.1007/s12149-016-1135-2. 10.1007/s12149-016-1135-2 27812791

[46] Barrington SF, Kluge R. FDG PET for therapy monitoring in Hodgkin and non-Hodgkin lymphomas. Eur J Nucl med Mol Imaging. 2017. doi:10.1007/s00259-017-3690-8. 10.1007/s00259-017-3690-8 PMC5541086 28411336

[47] Quak E, Hovhannisyan N, Lasnon C, Fruchart C, Vilque JP, Musafiri D (2014). The importance of harmonizing interim positron emission tomography in non-Hodgkin lymphoma: focus on the Deauville criteria. Haematologica.

[48] Kuhnert G, Boellaard R, Sterzer S, Kahraman D, Scheffler M, Wolf J, et al. Impact of PET/CT image reconstruction methods and liver uptake normalization strategies on quantitative image analysis. Eur J Nucl med Mol Imaging. 2015; doi:10.1007/s00259-015-3165-8. 10.1007/s00259-015-3165-8 26280981

[49] Barrington SF, Kluge R. FDG-PET for therapy monitoring in Hodgkin and non-Hodgkin lymphoma. Eur J Nucl Med Mol Imaging. 2017. doi:10.1007/s00259-017-3690-8. 10.1007/s00259-017-3690-8 PMC5541086 28411336

[50] Pinker K, Riedl C, Weber WA. Evaluating tumor response with FDG PET: updates on PERCIST, comparison with EORTC criteria and clues to future developments. Eur J Nucl med Mol Imaging. 2017; doi:10.1007/s00259-017-3687-3. 10.1007/s00259-017-3687-3 PMC5542859 28361188

[51] Quak E, Le Roux PY, Lasnon C, Robin P, Hofman MS, Bourhis D (2016). Does PET SUV harmonization affect PERCIST response classification?. J Nucl med.

[52] Lasnon C, Le Roux PY, Quak E, Robin P, Hofman MS, Bourhis D, et al. EORTC PET response criteria are more influenced by reconstruction inconsistencies than PERCIST, but both benefit from the EARL harmonization program. EJNMMI Phys. 2017;4(1):17. doi:10.1186/s40658-017-0185-4. 10.1186/s40658-017-0185-4 PMC5449363 28560574

[53] Lasnon C, Enilorac B, Popotte H, Aide N. Impact of the EARL harmonization program on automatic delineation of metabolic active tumour volumes (MATVs). EJNMMI Res. 2017;7(1):30. doi:10.1186/s13550-017-0279-y. 10.1186/s13550-017-0279-y PMC5374086 28361349

[54] Desseroit MC, Visvikis D, Tixier F, Majdoub M, Perdrisot R, Guillevin R (2016). Development of a nomogram combining clinical staging with (18)F-FDG PET/CT image features in non-small-cell lung cancer stage I-III. Eur J Nucl med Mol Imaging.

[55] Hatt M, Tixier F, Pierce L, Kinahan PE, Le Rest CC, Visvikis D (2017). Characterization of PET/CT images using texture analysis: the past, the present... Any future?. Eur J Nucl med Mol Imaging.

[56] Lovinfosse P, Janvary ZL, Coucke P, Jodogne S, Bernard C, Hatt M (2016). FDG PET/CT texture analysis for predicting the outcome of lung cancer treated by stereotactic body radiation therapy. Eur J Nucl med Mol Imaging.

[57] van Velden FH, Kramer GM, Frings V, Nissen IA, Mulder ER, de Langen AJ (2016). Repeatability of Radiomic features in non-small-cell lung cancer [(18)F]FDG-PET/CT studies: impact of reconstruction and delineation. Molecular Imaging and Biology : MIB : the Official Publication of the Academy of Molecular Imaging.

[58] Lasnon C, Majdoub M, Lavigne B, Do P, Madelaine J, Visvikis D (2016). 18F-FDG PET/CT heterogeneity quantification through textural features in the era of harmonisation programs: a focus on lung cancer. Eur J Nucl med Mol Imaging.

[59] Lasnon C, Houdu B, Kammerer E, Salomon T, Devreese J, Lebasnier A (2016). Patient's weight: a neglected cause of variability in SUV measurements? A survey from an EARL accredited PET centre in 513 patients. Eur J Nucl med Mol Imaging.

[60] Wahl RL, Jacene H, Kasamon Y, Lodge MA (2009). From RECIST to PERCIST: evolving considerations for PET response criteria in solid tumors. J Nucl med.

[61] Erselcan T, Turgut B, Dogan D, Ozdemir S (2002). Lean body mass-based standardized uptake value, derived from a predictive equation, might be misleading in PET studies. Eur J Nucl med Mol Imaging.

[62] Chowdhury B, Sjostrom L, Alpsten M, Kostanty J, Kvist H, Lofgren R (1994). A multicompartment body composition technique based on computerized tomography. International Journal of Obesity and Related Metabolic Disorders : Journal of the International Association for the Study of Obesity.

[63] Kim WH, Kim CG, Kim DW (2012). Comparison of SUVs normalized by lean body mass determined by CT with those normalized by lean body mass estimated by predictive equations in normal tissues. Nucl med Mol Imaging.

[64] Makris NE, Boellaard R, Visser EP, de Jong JR, Vanderlinden B, Wierts R (2014). Multicenter harmonization of 89Zr PET/CT performance. J Nucl med.

[65] FDG-PET/CT Technical Committee. FDG-PET/CT as an imaging biomarker measuring response to cancer therapy, version 1.05, Publicly Reviewed Version. QIBA. 2013. https://www.rsna.org/uploadedFiles/RSNA/Content/Science_and_Education/QIBA/QIBA_FDG-PET_Profile_v105_Publicly_Reviewed_Version_FINAL_11Dec2013.pdf.; 2015.

[66] Boellaard R, Quick HH (2015). Current image acquisition options in PET/MR. Semin Nucl med.

